# Public Restroom Access and Mental Health Among Gender-Minoritized Individuals in China

**DOI:** 10.1001/jamanetworkopen.2024.10546

**Published:** 2024-05-03

**Authors:** Yuanyuan Wang, Dongyu Liu, Meng Han, Jiaqi Li, Hui Yu

**Affiliations:** 1 Key Laboratory of Brain, Cognition and Education Sciences, Ministry of Education, China; School of Psychology, Center for Studies of Psychological Application, and Guangdong Key Laboratory of Mental Health and Cognitive Science, South China Normal University, Guangzhou, China; 2 Vanke School of Public Health, Tsinghua University, Beijing, China; 3 Division of Psychology, Faculty of Health and Life Sciences, De Montfort University, Leicester, United Kingdom

## Abstract

This cross-sectional study assesses the adequacy of gender-neutral public restrooms and examines the association of public restroom–related stress with mental health among gender-diverse individuals in China.

## Introduction

The transgender, nonbinary, and gender-diverse (henceforth, transgender) population faces unique and hostile stressors that are related to their minoritized identity.^1,2^ In particular, structural barriers transgender individuals face have manifested in sex-segregated public facilities, especially in public restrooms.^3,4^ Previous research revealed that transgender individuals whose appearance is incongruent with the ideal gender appearance often experience verbal, physical, and sexual harassment in public restrooms,^3^ leading to severe physical and mental health conditions.^4^

This marks the largest nationwide survey conducted in mainland China to explore public restroom accessibility and its impact on the mental health of the transgender population. Our objectives were 3-fold: (1) assess the adequacy of gender-neutral public restrooms for the transgender population, (2) explore the potential heterogeneity in restroom discrimination and mental health outcomes across transgender subgroups, and (3) examine the association of public restroom–related stress with mental health.

## Methods

This cross-sectional study is part of the 2021 China Transgender Health Survey, covering all 31 provinces, autonomous regions, and cities in mainland China. It was approved by the ethics committee of The Second Xiangya Hospital of Central South University. Gender identity, public restroom experiences, and mental health outcomes (anxiety, depression, posttraumatic stress, suicidality, and self-harm) were evaluated using validated self-report inventories (details available in the eAppendix in Supplement 1).

Statistical analyses were conducted from July 2022 to March 2024 via R statistical software version 4.3.2 (R Project for Statistical Computing). Between-group comparisons among transgender men, transgender women, nonbinary-genderqueer individuals, and others were conducted by χ^2^ tests on categorical variables, and nonparametric analysis of variance Kruskal-Wallis tests on continuous scores. Multivariate regression analyzed mental health outcomes estimated by verbal, physical, and sexual abuse in public restrooms, and the consequences of avoiding public restrooms. Significance was set at α < .05.

## Results

Of 9161 participants who provided consent and completed the survey, 7576 (82.7%) constituted the final sample. Of the 7576 participants (mean [SD] age, 15.6 [5.15] years), 4147 (54.7%) were assigned male at birth and 3427 (45.2%) were assigned as female. As shown in Table 1, 2170 participants (28.6%) reported access to gender-neutral restrooms, yet 5760 (76.0%) preferred them. Hostile treatment in public restrooms was reported, including 741 participants (9.8%) experiencing verbal abuse, 83 (1.1%) experiencing physical abuse, and 229 (3.0%) experiencing sexual abuse. A substantial number of participants surpassed the clinical diagnosis thresholds for anxiety (2670 participants [35.2%]), depression (3214 participants [42.4%]), and posttraumatic stress disorder (3805 participants [50.2%]). Moreover, 3210 (42.4%) reported suicide attempts, and 2333 (30.8%) engaged in self-harm. Notably, transgender women reported the most adverse mental health in between-group comparisons. As shown in Table 2, the multivariate regression model revealed that verbal abuse (β, 0.11-0.14), sexual abuse (β, 0.16-0.42), and negative consequences of avoiding public restrooms (β, 0.09-0.13) were significantly associated with increased levels of anxiety, depression, posttraumatic stress, suicidality, and self-harm.

**Table 1.** Characteristics of the Study Population and Responses to Survey Items

Characteristic	Participants, No. (%)					χ^2^ or Kruskal-Wallis test^a^	FDR-adjusted *P* value
	Overall (N = 7576)	Transgender men (n = 1453)	Transgender women (n = 2824)	Nonbinary or genderqueer (n = 2078)	Others (n = 1221)		
Age group, y							
≤17^b^^,^^c^^,^^d^^,^^e^	5556 (73.3)	996 (68.5)	2109 (74.7)	1593 (76.7)	858 (70.3)	48.44	<.001
18-24^b^^,^^c^	434 (5.7)	346 (23.8)	559 (19.8)	406 (19.5)	275 (22.5)		
≥25^b^^,^^c^^,^^e^^,^^f^	1586 (20.9)	111 (7.6)	156 (5.5)	79 (3.8)	88 (7.2)		
Sex assigned at birth							
Male^b^^,^^c^^,^^d^^,^^f^^,^^g^	4147 (54.7)	0	2824 (100)	709 (34.1)	614 (50.3)	4459.2	<.001
Female^b^^,^^c^^,^^d^^,^^e^^,^^f^^,^^g^	3427 (45.2)	1452 (99.9)	0	1368 (65.8)	607 (49.7)		
Others	2 (0)	1 (0.1)	0	1 (0.0)	0		
Ethnicity							
Han^f^	7013 (92.6)	1335 (91.9)	2647 (93.7)	1900 (91.4)	1131 (92.6)	10.46	.02
Others^f^	563 (7.4)	118 (8.1)	177 (6.3)	178 (8.6)	90 (7.4)		
Education							
Below high school^b^^,^^e^^,^^f^	514 (6.8)	125 (8.6)	209 (7.4)	94 (4.5)	86 (7.0)	71.76	<.001
High school or equivalent^d^^,^^f^	1829 (24.1)	348 (24.0)	760 (26.9)	446 (21.5)	275 (22.5)		
University degree or equivalent^c^^,^^f^	4647 (61.3)	851 (58.6)	1693 (60.0)	1345 (64.7)	758 (62.1)		
Graduate degree^b^^,^^d^^,^^f^	586 (7.7)	129 (8.9)	162 (5.7)	193 (9.3)	102 (8.4)		
Religion							
Yes^b^^,^^d^^,^^f^	784 (10.3)	167 (11.5)	241 (8.5)	225 (10.8)	151 (12.4)	17.95	.001
No^b^^,^^d^^,^^f^	6792 (89.7)	1286 (88.5)	2583 (91.5)	1853 (89.2)	1070 (87.6)		
Gender-neutral restroom access							
Yes^b^^,^^c^^,^^d^^,^^e^	2170 (28.6)	331 (22.8)	921 (32.6)	638 (30.7)	280 (22.9)	101.91	<.001
No^b^^,^^c^^,^^f^^,^^g^	3392 (44.8)	739 (50.9)	1184 (41.9)	945 (45.5)	524 (42.9)		
Unsure^d^^,^^e^^,^^g^	2014 (26.6)	383 (26.4)	719 (25.5)	495 (23.8)	417 (34.2)		
Gender-neutral restroom ever used							
Yes^b^^,^^c^^,^^d^^,^^e^^,^^f^^,^^g^	2444 (32.3)	398 (27.4)	1087 (38.5)	702 (33.8)	257 (21.0)	138.38	<.001
No^b^^,^^c^^,^^d^^,^^e^^,^^f^^,^^g^	5132 (67.7)	1055 (72.6)	1737 (61.5)	1376 (66.2)	964 (79.0)		
Gender-neutral restroom preference							
Yes^b^^,^^d^^,^^e^^,^^f^^,^^g^	5760 (76.0)	1203 (82.8)	2283 (80.8)	1517 (73.0)	757 (62.0)	214.73	<.001
No^b^^,^^d^^,^^e^^,^^f^^,^^g^	1816 (24.0)	250 (17.2)	541 (19.2)	561 (27.0)	464 (38.0)		
Verbal abuse in public restroom							
Yes^b^^,^^c^^,^^d^^,^^e^^,^^f^	741 (9.8)	223 (15.3)	268 (9.5)	183 (8.8)	67 (5.5)	79.04	<.001
No^b^^,^^c^^,^^d^^,^^e^^,^^f^	6835 (90.2)	1230 (84.70)	2556 (90.5)	1895 (91.2)	1154 (94.5)		
Physical abuse in public restroom							
Yes^g^	83 (1.1)	11 (0.8)	47 (1.7)	12 (0.6)	13 (1.1)	15.13	.002
No^g^	7493 (98.9)	1442 (99.20)	2777 (98.3)	2066 (99.4)	1208 (98.9)		
Sexual abuse in public restroom							
Yes^b^^,^^c^^,^^g^	229 (3.0)	22 (1.5)	101 (3.6)	58 (2.8)	48 (3.9)	18.05	.002
No^b^^,^^c^^,^^g^	7347 (97.0)	1431 (98.50)	2723 (96.4)	2020 (97.2)	1173 (96.1)		
Anxiety							
Score, mean (SD)^b^^,^^c^^,^^d^^,^^f^^,^^g^	8.06 (5.82)	7.09 (5.75)	8.73 (5.86)	7.90 (5.76)	7.92 (5.71)	90.08	<.001
Score ≥10^b^^,^^c^^,^^d^^,^^f^^,^^g^	2670 (35.2)	423 (29.1)	1123 (39.8)	704 (33.9)	420 (34.4)	51.32	<.001
Depression							
Score, mean (SD)^b^^,^^c^^,^^d^^,^^f^^,^^g^	14.3 (7.22)	12.8 (7.51)	15.5 (7.09)	14.1 (7.14)	14.0 (6.90)	139.08	<.001
Score ≥17^b^^,^^c^^,^^d^^,^^f^^,^^g^	3214 (42.4)	496 (34.1)	1387 (49.1)	847 (40.8)	484 (39.6)	98.85	<.001
Posttraumatic stress disorder							
Score, mean (SD)^b^^,^^c^^,^^e^^,^^f^^,^^g^	5.23 (3.34)	4.64 (3.41)	5.53 (3.30)	5.12 (3.34)	5.43 (3.26)	71.54	<.001
Score ≥6^b^^,^^c^^,^^f^^,^^g^	3805 (50.2)	617 (42.5)	1536 (54.4)	1016 (48.9)	636 (52.1)	57.78	<.001
Suicidality							
Score, mean (SD)^b^^,^^c^^,^^d^^,^^e^^,^^f^	9.74 (7.45)	8.75 (7.45)	11.1 (7.40)	9.42 (7.29)	8.37 (7.34)	161.95	<.001
Life-time suicide attempt^b^^,^^d^^,^^e^^,^^f^^,^^g^	3210 (42.4)	568 (39.1)	1405 (49.8)	811 (39.0)	426 (34.9)	106.91	<.001
Nonsuicidal self-injury							
Mean (SD)^b^^,^^c^^,^^d^^,^^e^^,^^f^	6.84 (10.6)	5.97 (9.84)	6.97 (10.6)	7.79 (11.5)	5.98 (10.1)	25.67	<.001
Self-harm in past 12 mo^b^^,^^c^^,^^d^^,^^e^	2333 (30.8)	364 (25.1)	940 (33.3)	701 (33.7)	328 (26.9)	47.99	<.001

Abbreviation: FDR, false discovery rate.

^a^ χ^2^ tests were conducted for categorical variables, and Kruskal-Wallis tests were conducted for numeric variables.

^b^ Follow-up 2-group comparisons were conducted and the results were labeled with significant difference between transgender man (ie, individuals assigned female at birth but identifying as a man or transgender man) and transgender woman (ie, individuals assigned male at birth but identifying as a woman or transgender woman).

^c^ Follow-up 2-group comparisons were conducted and the results were labeled with significant difference between transgender man and nonbinary or genderqueer (ie, individuals who believed their gender identities could not be defined on a binary scale).

^d^ Follow-up 2-group comparisons were conducted and the results were labeled with significant difference between transgender woman and others (including cross-dresser, uncertain, and none of the above).

^e^ Follow-up 2-group comparisons were conducted and the results were labeled with significant difference between nonbinary or genderqueer and others.

^f^ Follow-up 2-group comparisons were conducted and the results were labeled with significant difference between transgender woman and nonbinary or genderqueer.

^g^ Follow-up 2-group comparisons were conducted and the results were labeled with significant difference between transgender man and others.

**Table 2.** Multivariate Linear Regression Estimates of Mental Health Outcomes Based on Stress Related to Public Restroom Usage (N = 7576)^a^

Variable	β (95% CI)				
	Anxiety	Depression	PTSD	Suicidality	NSSI
Verbal abuse	0.14 (0.07 to 0.22)^b^	0.11 (0.04 to 0.19)^c^	0.12 (0.04 to 0.20)^c^	0.11 (0.03 to 0.18)^c^	0.12 (0.04 to 0.19)^c^
Physical abuse	0.16 (−0.06 to 0.39)	0.11 (−0.11 to 0.33)	0.16 (−0.07 to 0.38)	0.20 (−0.02 to 0.42)	0.22 (0.00 to 0.43)
Sexual abuse	0.26 (0.13 to 0.40)^b^	0.16 (0.03 to 0.30)^d^	0.42 (0.29 to 0.55)^b^	0.32 (0.19 to 0.45)^b^	0.25 (0.11 to 0.38)^b^
Avoid consequence	0.13 (0.10 to 0.15)^b^	0.12 (0.10 to 0.14)^b^	0.12 (0.09 to 0.14)^b^	0.13 (0.11 to 0.16)^b^	0.09 (0.07 to 0.11)^b^
Adjusted *R^2^*	.026	.020	.027	.028	.016

Abbreviations: PTSD, posttraumatic stress disorder; NSSI, nonsuicidal self-injury.

^a^ A multivariate regression model was constructed using the lavaan package in R, which includes 5 outcome variables (anxiety, depression, PTSD, suicidality, and NSSI, with the scores of anxiety and self-injury being square rooted), estimated by 4 independent variables (verbal abuse, physical abuse, sexual abuse experienced in public restrooms, and consequences of avoiding public restrooms), while controlling for age, birth-assigned sex, ethnicity, education, and religion as covariates. Further details regarding the modeling descriptions can be found in the eAppendix in Supplement 1.

^b^ *P* < .001.

^c^ *P* < .01.

^d^ *P* < .05.

## Discussion

The findings of this cross-sectional study revealed that transgender individuals encountered restroom hostility and often avoided public restrooms. These harmful experiences were significantly associated with increased levels of anxiety, depression, posttraumatic stress, suicidality and self-harm. Notably, transgender women appeared to report the most adverse mental health outcomes.

Our data indicate that transgender individuals show a preference for gender-neutral restrooms when available, but the availability of gender-neutral restrooms remains extremely limited in China. Evidently, a better economic condition is linked to improved facilities, increased acceptance, and reduced discrimination for the transgender community.^5^ Access to restrooms, a seemingly mundane aspect of daily life, becomes a major stressor to transgender individuals, damaging their mental resilience.^6^ The cross-sectional design of this study limits establishing causality between public restroom access and mental health outcomes. Still, it is crucial to reduce the structural inequalities experienced by the transgender community, recognize inadequacies in public facilities, and prioritize the physical and mental well-being of society.

## Notes

Supplement 1. **eAppendix.** Supplemental Methods
eReferences

Supplement 2.
Data Sharing Statement
