# Supplementary material for: Public Restroom Access and Mental Health Among Gender-Minoritized Individuals in China
Source: JAMA Netw Open. 2024 May 3;7(5):e2410546. doi: 10.1001/jamanetworkopen.2024.10546 (PMC11069084; doi:10.1001/jamanetworkopen.2024.10546)
Supplement: Supplement 1. — eAppendix. Supplemental Methods eReferences [file jamanetwopen-e2410546-s001.pdf]

Supplemental Online Content

Wang Y, Liu D, Han M, Li J, Yu H. Public restroom access and mental health among gender-minoritized individuals in China. *JAMA Netw Open*. 2024;7(5):e2410546.
doi:10.1001/jamanetworkopen.2024.10546

eAppendix. Supplemental Methods
eReferences

This supplemental material has been provided by the authors to give readers additional information about their work.

© 2024 Wang Y et al. *JAMA Network Open.*

eAppendix. Supplemental Methods

Reporting style and participant screening

The reporting of the study follows the Strengthening the Reporting of Observational Studies in Epidemiology (STROBE) guidelines for cross-sectional studies. A total of 9,161 participants completed the survey, from which 393 were excluded, due to incorrect responses to attention questions (n = 268) or repeated submissions and unreasonable age (n = 125). Furthermore, 1,192 participants were excluded after scrutinizing the consistency between answers regarding sex assigned at birth and gender identity. Specifically, individuals assigned male at birth who identified as men were considered potentially cisgender (n = 519), as were those assigned female at birth who identified as women (n = 673).

Measures of Mental Health Outcome Variables

Anxiety was measured by the 7-item Generalized Anxiety Disorder Scale, GAD-7 [1]. The Cronbach's α was 0.92 in this sample. A total score (ranging from 0-21) of 10 or more indicates moderate to severe anxiety symptoms [1].

Depression was measured by 9 items adopted from the Centre for Epidemiologic Studies Depression Scale (CES-D) [2]. The Cronbach's α was 0.91. A total score (ranging from 0-27) of 17 or more indicates a severe risk of depression symptoms [3].

Post-traumatic Stress was measured by the 10-item Trauma Screening Questionnaire, TSQ [4]. The Cronbach's α was 0.87. The total score ranged from 0-10, and 6 or more indicates moderate to severe PTSD symptoms [5].

Suicidality was measured by making participants respond to four statements with Yes or No [6]. 1) If they have never considered suicide, their total score was 0; if yes, they received a score of 1. 2) If they had suicidal thoughts within the last 12 months, an additional 3 points were added. 3) If they had ever had a suicidal plan, they received an additional 5 points. 4) If they have ever attempted suicide, they received an additional 10 points. The suicide total score ranged from 0 to 19.

Non-suicidal self-injury (NSSI) was measured by two items: 1) If they have never conducted self-harm, their total score was 0; if yes, they scored 1 on this item. If it was more than 12 months ago, they received 3 points; if within the last 12 months, 5 points. 2) the frequency of NSSI within the past 12 months was also measured. They were given a score that was double the number of times they self-harmed. However, if they self-harmed more than 10 times, they were given an additional 30 points. The self-harm score ranged from 0 to 39.

Statistical analysis

Objective 1 was evaluated by inquiring about the availability, preference, and use of gender-neutral restrooms in participants' areas. Detailed results are presented in Table 1.

Objective 2 involved between-group comparisons among the four transgender sub-groups. Chi-square tests were employed for categorical variables, and non-parametric ANOVA Kruskal-Wallis tests for continuous scores, with findings presented in Table 1.

Objective 3 was evaluated using a multivariate regression model, with results detailed in Table 2.

Firstly, the "moments" package in R was used to test the skewness of the distribution for all five outcome variables. Raw data inspection revealed an approximately symmetrical distribution of depression, post-traumatic stress, and suicidality (skewness <0.5), while anxiety was moderately skewed (skewness=0.53), and self-injury was highly skewed (skewness=1.89) respectively. Consequently, square root transformations were performed on anxiety and self-injury scores.

Utilizing the "lavaan" package in R, we constructed a multivariate regression model, including five outcome variables (anxiety, depression, post-traumatic stress, suicidality, and self-injury, with anxiety and self-injury scores transformed), predicted by four independent variables (verbal abuse, physical abuse, sexual abuse experienced in public restrooms, and consequences of avoiding public restrooms). In addition, basic demographic information including age, birth-assigned sex, ethnicity, education, and religion were controlled for as covariates. To obtain the standardized beta coefficients, z-scores were calculated for all numeric variables in the model.

© 2024 Wang Y et al. *JAMA Network Open.*

eReferences

1. Spitzer RL, Kroenke K, Williams JB, Löwe B. A brief measure for assessing generalized anxiety disorder: the GAD-7. *Archives of internal medicine*. 2006;166(10):1092-1097. doi:10.1001/archinte.166.10.1092
2. Radloff LS. The CES-D scale: A self-report depression scale for research in the general population. *Applied psychological measurement*. 1977;1(3):385-401. doi:10.1177/014662167700100306
3. Wang Y, Han M, Zhang Y, et al. A national transgender health survey from China assessing gender identity conversion practice, mental health, substance use and suicidality. *Nature Mental Health*. 2023;1(4):254-265. doi: 10.1038/s44220-023-00041-z
4. Brewin CR, Rose S, Andrews B, et al. Brief screening instrument for post-traumatic stress disorder. *Br J Psychiatry*. Aug 2002; 181:158-62. doi:10.1017/s0007125000161896
5. Walters JT, Bisson JI, Shepherd JP. Predicting post-traumatic stress disorder: validation of the Trauma Screening Questionnaire in victims of assault. *Psychological medicine*. 2007;37(1):143-150. doi: 10.1017/S0033291706008658
6. Wang Y, Yu H, Yang Y, et al. Mental health status of cisgender and gender-diverse secondary school students in China. *JAMA network open*. 2020;3(10):e2022796-e2022796. doi:10.1001/jamanetworkopen.2020.22796

© 2024 Wang Y et al. *JAMA Network Open.*
