# Supplementary material for: Public Restroom Access and Mental Health Among Gender-Minoritized Individuals in China
Source: JAMA Netw Open. 2024 May 3;7(5):e2410546. doi: 10.1001/jamanetworkopen.2024.10546 (PMC11069084; doi:10.1001/jamanetworkopen.2024.10546)
Supplement: Supplement 2. — Data Sharing Statement [file jamanetwopen-e2410546-s002.pdf]

Data Sharing Statement

Wang. Public Restroom Access and Mental Health Among Gender-Minoritized Individuals in China. *JAMA Netw Open*. Published May 03, 2024. doi:10.1001/jamanetworkopen.2024.10546

Data
Data available: No

Additional Information
Explanation for why data not available: Data will be made available only to potential collaborators with ethical approval after they submit a research proposal application by contacting the corresponding author.
